# Mortality Risk Associated with Diabetic Foot Complications in People with or without History of Diabetic Foot Hospitalizations

**DOI:** 10.3390/jcm11092454

**Published:** 2022-04-27

**Authors:** Piergiorgio Francia, Elisa Gualdani, Laura Policardo, Leonardo Bocchi, Flavia Franconi, Paolo Francesconi, Giuseppe Seghieri

**Affiliations:** 1 Department of Information Engineering, University of Florence, 50139 Florence, Italy; leonardo.bocchi@unifi.it; 2 Epidemiology Unit, Agenzia Regionale Sanità, 50141 Florence, Italy; elisa.gualdani@ars.toscana.it (E.G.); laura.policardo@gmail.com (L.P.); paolo.francesconi@ars.toscana.it (P.F.); gseghier@tin.it (G.S.); 3 Laboratorio Nazionale di Farmacologia e Medicina di Genere, Istituto Nazionale Biostrutture Biosistemi, University of Sassari, 07100 Sassari, Italy; franconi@uniss.it

**Keywords:** diabetic foot complications, mortality risk, hospitalizations, first ever incident diabetic foot hospital admission, amputations, diabetic foot ulcers

## Abstract

The aim of this study was to evaluate the risk of death after hospitalizations for diabetic foot (DF) complications, comparing two different cohorts of people with or without a prior history of DF hospitalizations across the years 2011 to 2018 in Tuscany, Italy. The DF complications were categorized by administrative source datasets such as: amputations (both major and minor), gangrene, ulcers, infections, Charcot and revascularizations. A further aim was to present the trend over time of the first ever incidents of diabetic foot hospitalizations in Tuscany. The eight-year-mortality rate was higher in the cohort with prior hospitalizations (*n* = 6633; 59%) compared with the cohort with first incident DF hospitalizations (*n* = 5028; 44%). Amputations (especially major ones) and ulcers had the worst effect on survival in people without basal history of DF hospitalizations and respectively in those with a history of prior DF hospitalizations. In both cohorts, revascularization procedures, when compared to ulcers, were associated with a significantly reduced risk of mortality. The prevalence rate of minor amputations showed a slightly rising trend over time. This result agrees with the national trend. Conversely, the progressive increase over time of revascularizations, associated with the fractional decrease in the rate of gangrene, suggests a trend for more proactive behavior by DF care teams in Tuscany.

## 1. Introduction

Diabetic foot (DF) is a leading cause of disability; it represents huge costs for healthcare systems and early mortality in people with diabetes [1,2,3,4,5,6,7,8,9,10]. A point which remains to be more extensively addressed is evaluating the different impact on death risk after a first ever incident of diabetic foot hospital admission for DF complications compared to the risk for people who experienced a previous DF hospitalization: all this would help to better understand the natural history of DF, its costs and, consequently, the resources to be allocated for care or prevention of DF and its complications. A recent paper, for instance, has shown that lower limb amputations are among the most expensive complications of diabetes and that their cost is significantly high, particularly after a first ever incident of diabetic foot hospitalization [11]. In addition, while among all DF complications amputations and diabetic foot ulcers seem to be associated with a higher risk of mortality [12,13], evidence has also been accumulated over time that revascularization procedures, which have an important role in the strategy of anatomical and functional rescue of lower limbs, protect from premature death [14,15]. To better elucidate these issues, we have carried out a retrospective observational study using administrative data sources regarding death incidence related to hospitalizations for DF in the region of Tuscany, in central Italy, over the years 2011 to 2018. The first aim of the present study was to evaluate the trend of first incident hospitalizations due to each DF complication across the entire period of eight years in this population. A further goal was grading the risk of death associated with each single foot lesion, as diagnosed from hospital discharges in two different cohorts: in people with prevailing DF lesions at basal as testified by the history of prior DF hospitalizations, and in those who were hospitalized for diabetic foot complications for the first time.

## 2. Materials and Methods

### 2.1. Study Design and Data Source

The population under study consisted of all identified people with diabetes residing in Tuscany, a region of central Italy, as of 1 January 2011, retrospectively followed up until 31 December 2018. The diagnosis of diabetes was based on a validated algorithm by utilizing administrative databases at the Regional Health Agency of Tuscany, in Florence, Italy, as previously detailed [16]. Such regional dataset has been validated and shown to cover more than 80% of all diabetic patients living in Tuscany [17]. This initial population was divided into two cohorts: the first including all individuals who had no previous hospitalizations for DF complications as of 1 January 2011, or at entry into the study. The second cohort included all individuals with a history of previous DF hospitalizations at baseline.

### 2.2. Definition and Classification of DF Complications

DF hospitalizations were recorded according to any of the following ICD-9 CM codes: ulcers: 440.23, 707.14, 707.15; Charcot neuro-arthropathy: 713.0, 713.5, 713.8; infections: 6811, 6819, 6826, 6827, 6829, 730.07, 730.17, 730.27, 99.21; gangrene: 440.20, 440.21, 440.22, 440.23, 440.29, 443.9, 785.4, 440.0, 440.24; major and minor lower extremity amputations: 84.10–84.19; revascularizations (surgical: 39.25, 39.29; endoluminal: 39.50, 39.90). In both cohorts, the presence of co-morbidities was diagnosed according to the Charlson index. This index is an integrated indicator referring co-morbidities as from all previously hospital discharges [18] and scored as 0, 1 or 2, thus reflecting the increase in their complexity and severity.

### 2.3. Outcomes and Statistical Procedures

The incidence of death (all-cause mortality) occurring within the period 1 January 2011 to 31 December 2018 was retrieved in both cohorts from the database of the regional registry office. Time to event was considered as the interval from the first ever incident of diabetic foot hospitalizations or from 1 January 2011 to death or to end of study, and survival rates were determined through Kaplan-Meier curves.

After testing for proportionality of risks, the Cox proportional hazards model has been used to assess the hazard ratios (HRs) of all-cause death after any incidental first-ever DF complication in a model where foot ulcers were the reference group and after adjusting for Charlson index, sex, age and antidiabetic therapy. In this cohort, the incidence rates of DF complications across the entire period 2011–2018 was calculated by trend test after chi-square. Among those with prevailing DF hospitalizations at basal, death HRs were calculated by means of Cox proportional hazards models, and the time to event was considered as the interval from 1 January 2011 to death or to end of study, after adjusting for the same covariates and with foot ulcer as the reference group.

All data were anonymized and based on administrative datasets, preventing any disclosure of patients’ identity as well as of any other sensitive information. Because of such formal protection, no informed consent or any approval by an Ethics Committee was required, according to current national and regional rules.

All analyses were performed using SAS ver. 9.3, SAS Institute Inc., Cary, NC, USA.

## 3. Results

**Figure 1:**
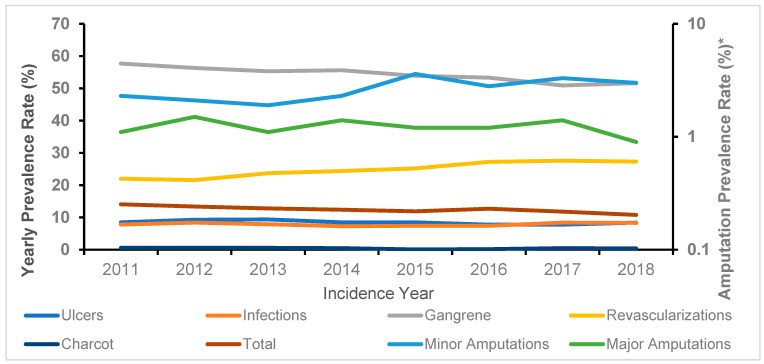
Prevalence rates of hospitalizations for diabetic foot complications across the years 2011 to 2018 in Tuscany (* Logarithmic scale).

**Figure 2:**
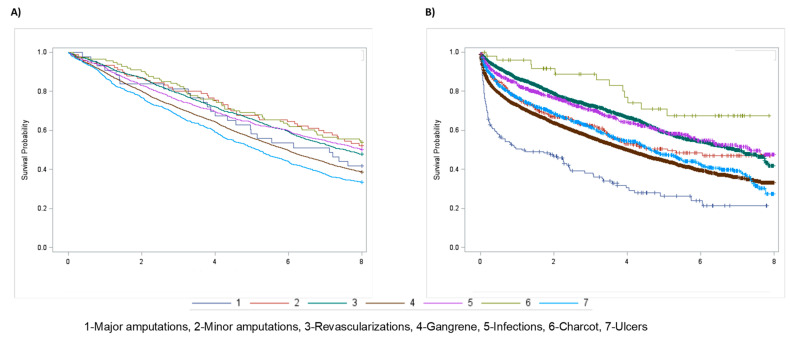
Survival probability by Kaplan Meier analysis for diabetic foot complications in people with previous hospitalizations for diabetic foot (**A**) and after first incident hospitalization for diabetic foot (**B**).

**Figure 3:**
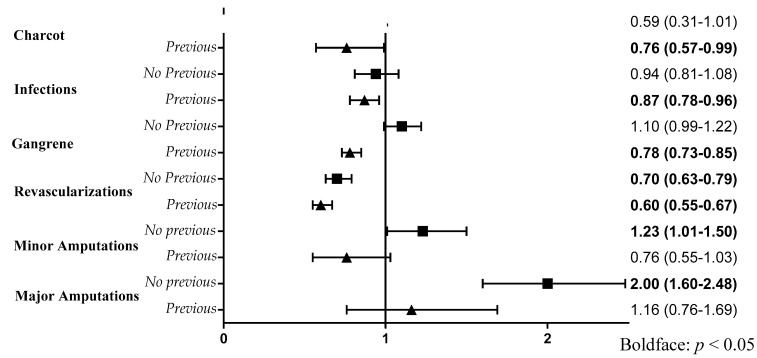
Adjusted Hazard Ratios (HR) of diabetic foot complications in both cohorts with (▲) and without (◾) previous hospitalizations for diabetic foot. Ulcers are considered here as the reference group.

The main characteristics of the two cohorts under study are reported in Table 1. Both cohorts contained about the same number of hospitalizations (11,246 vs. 11,529), and age was on average more advanced in those without prior DF hospitalizations (74 ± 10 yr vs. 71 ± 11 yr; *p* < 0.05). Males were more represented in both cohorts, even if with a preponderance significantly lower among those without previous hospitalizations at basal (61.6% vs. 66.1%; *p* < 0.05). Most first ever DF hospitalizations were due to revascularization procedures or to gangrene, with lower rates for amputations and Charcot. The incidence rate of ulcers was similar to that of infections: 22.3; 95% CI 21–23.8 per 1000 p-y vs. 20.4; 95% CI 21–23.8 per 1000 p-y. Comorbidities were more severe in those with prior DF hospitalization at baseline, with the percentage of Charlson index ≥2 approximately twice as high: 75.8% vs. 41.7%; *p* = 0.0001. Therapy with insulin was about twice as prevalent in the cohort with prior DF hospitalizations at baseline as compared with the cohort with first incident hospitalizations. In the cohort without prior hospitalizations for diabetic foot, there were 5028 deaths with a 56% survival rate at the end of follow-up. Instead, in the cohort with prior hospitalizations, there were 6633 deaths with a survival rate at follow-up of 41%; *p* < 0.05. The rate of deaths was higher after any first incident hospitalized complication, especially after both major or minor amputations and gangrene. However, revascularizations and ulcers had approximately the same mortality incidence rate in the two cohorts (0.40; 95% CI 0.37–0.42 per 1000 p-y vs. 0.39; 95% CI 0.37–0.42 per 1000 p-y for revascularizations and 0.67; 95% CI 0.60–0.74 per 1000 p-y vs. 0.72; 95% CI 0.67–0.77 per 1000 p-y for ulcers). The prevalence rates for any diabetic foot complications, evaluated by trend test after chi-square across the total eight-year period, showed no significant trend, except for the curve of gangrene, which had a negative slope (Figure 1; Table 2). The rates of minor amputations and revascularizations increased over the entire period; trend test: *p* < 0.0001 for both. Survival analysis estimated by Kaplan-Meier curves showed that in the cohort without prior hospitalizations, major amputations had the worst survival rate over time, while in the cohort with prevalent diabetic foot complications at baseline, ulcers were associated with the poorest prognosis (Figure 2). Both major and minor amputations showed a significantly higher risk of death only in the cohort without previous hospitalizations. This was verified after calculating adjusted HRs of death through Cox regression models, considering ulcers as the reference group (Figure 3). Ulcers had a worse prognosis compared to gangrene, infections and Charcot in the cohort with prevalent diabetic foot at baseline. Revascularizations had a protective effect against mortality by about 30–40% in both cohorts.

## 4. Discussion

Diabetic foot is associated with a significant increase in the risk of premature death [3,4,5,6,7,8,9,10,11,12,19]. It has, indeed, been rightly said that the reduction in life expectancy of patients with diabetic foot can be compared to that of those affected with cancer [3]. The main purpose of the present study was to better define the role played by different DF complications in increasing the risk of mortality, comparing two different cohorts of people: those with or without prior hospitalizations for DF complications. The patients were followed retrospectively for eight years (2011–2018) in Tuscany, an Italian region that has about 3.5 million people. A further matter in question was to verify what role the procedures of revascularization played in eventually modifying the risk of death: a point that has not always been addressed by most prior studies. The yearly prevalence rate of first ever incidents of diabetic foot hospitalizations in Tuscany was substantially stable over time. The prevalence rate of major amputations was very low and remained unchanged over time. This result reflects, at least in part, the trend towards a continuous slight decline of major amputations in Italy in last decade [20]. The prevalence rate of minor amputations was slightly rising over time, and this is again in agreement with a similar national trend [20], while the progressive increase over time of revascularizations, associated with the fractional decrease in the rate of gangrene, suggests more proactive measures by diabetic foot care teams in our region, particularly targeted at procedures for the rescue of lower limbs in patients with more advanced vascular ischemic diseases. Both cohorts under study, with or without prior DF hospitalizations, were significantly different in several respects: the cohort with prior hospitalizations was younger, contained more males, had more comorbidities and was more frequently treated with insulin, while the adjusted risks of death for each complication appeared substantially more impacting after a first hospitalization more closely related to ischemic vascular complications such as amputations and gangrene. Regardless of DF complications, total mortality rates at 8 years were higher in the cohort with prior hospitalizations (59%) as compared with the mortality of the cohort counting first incident hospitalizations for DF (44%); *p* < 0.05. This agrees with the range of death incidence rates reported by previous epidemiological studies referring to cohorts with or without a history of DF [5,6,13,21,22]. It is, however, difficult to compare mortality rates across different countries, since most studies were designed to compare the risk of mortality between people with diabetic foot lesions and those without diabetic foot, and, additionally, many studies did not distinguish between first and recurrent hospitalizations. It is noteworthy, however, that the greatest risk of death was represented by ulcers in those with prior hospitalizations and by major amputations in patients experiencing a first ever hospitalization for DF. In this respect, a recent epidemiological study regarding the incidence of hospitalizations and of overall mortality in Piedmont, a region of northwestern Italy, clearly demonstrated that mortality risk was significantly higher after the new incident hospitalizations for both minor and major amputations of lower limbs, considered as the most selective expression of vascular DF [23]. The higher prevalence of women (39% vs. 34%) among first incident DF hospitalizations compared with those with DF at basal is in line with what was previously reported [24]. This study, moreover, shows that in the cohort with prior hospitalizations at basal, in the presence of the competing risk of premature death after amputations, diabetic foot ulcers appear as the lesions with the worst prognostic effect regarding survival, in agreement with what is widely reported by the literature [2,6,7]. A recent study has moreover demonstrated that ulcers are associated with a lower amputation-free survival rate [13], and, consequently, ulcers, especially ischemic ulcers, could significantly predict amputations in both cohorts, mediating by this way their final effect on death risk, even if the design of this study is not able to clarify this aspect. In addition, in those with prior hospitalizations for DF complications, it is interesting to note that even a classical ischemic lesion such as gangrene has a lesser mortality risk when compared to ulcers seemingly associated with both ischemic and non-ischemic pathogenesis [25]. Revascularizations, on the contrary, are characterized by a significant reduction in the overall risk of mortality compared to ulcers in both cohorts, further highlighting the importance of revascularization procedures, not only to save the functional integrity of the lower limbs but also to improve life expectancy in these patients. In this respect, interestingly, the positive effect of revascularization is evident not only when it represents a first event but also among those with a history of previous hospitalizations for DF.

### 4.1. Limitations and Strengths of the Study

As with all retrospective cohort studies based on administrative data, the main limitation of this study is that the lack of clinical data prevents a more thorough evaluation of the eventual interrelationships between death risk and severity of foot lesions. A further limitation is having considered only hospitalizations (both ordinary and day-hospital discharges), excluding other care settings, even if hospitalizations could reasonably include all more complicated clinical situations. The strength of our study may be found in the vast sample of the population involved and in the solid methods used to identify diabetes, as well as in the homogeneity in treatment of patients, as expected from a single regional public health system with free access to all resident citizens.

### 4.2. Conclusions

The prevalence rates for diabetic foot complications generally showed no significant trend, except for gangrene, which had a negative trend. Rates of minor amputations and revascularizations have instead increased throughout the entire period. The progressive increase over time for revascularizations, associated with the fractional decrease in the rate of gangrene, suggests a trend for more proactive measures by DF care teams, particularly targeted at procedures for the rescue of lower limbs in these patients. According to this study, moreover, the adjusted risk of mortality after hospitalizations for DF complications was completely different in cohorts with or without a prior history of hospitalized DF complications. Those without prior history showed an overall lesser percentage rate of deaths compared those with a prior history of hospitalizations due to complications. Amputations (especially major) had the worst effect on survival in people without a history of DF hospitalizations at basal. In those with a history of prior DF hospitalizations, ulcers predicted the worst prognosis. In both cohorts, revascularization procedures significantly reduced the risk of mortality. These peculiarities should be taken fully into consideration when evaluating data from epidemiological studies about the death risk associated with DF complications and could be useful for health care providers and policy makers.

## Figures and Tables

**Table 1:** Descriptive analysis for variables of both cohorts under study, with or without a prior history of hospitalizations for diabetic foot complications.

without Prior Hospitalizations for Diabetic Foot								
Diabetic Foot Lesions	Major Amputations	Minor Amputations	Revascularizations	Gangrene	Infections	Charcot	Ulcer	Total
**No. (%)**	143 (1.2)	306 (2.6)	2854 (24.7)	6282 (54.5)	908 (7.9)	51 (0.4)	985 (8.5)	**11,529**
**Incidence rate of first hospitalization per 1000 p-y (95%CI)**	3.0 (2.5–3.5)	6.5 (5.8–7.3)	77.3 (74.5–80.2)	293.2 (286–300.6)	20.4 (19.1–21.7)	1.1 (0.8–1.4)	22.3 (21.0–23.8)	**4.6 (4.5–4.7)**
**Mean Age yr (SD)**	74 (12)	68 (13)	70 (10)	73 (10)	66 (13)	62 (13)	72 (12)	**71 (11)**
**Male Sex No. (%)**	73 (51)	199 (65)	1875 (66)	3865 (54.4)	548 (60.3)	30 (58.8)	518 (52.6)	**61.6**
**Charlson index**								
0 No. (%)	46 (31.2)	145 (47.4)	1149 (40.3)	2364 (37.6)	435 (47.9)	22 (43.1)	413 (41.9)	**39.7**
1 No. (%)	22 (15.4)	54 (17.6)	520 (18.2)	1182 (18.8)	165 (18.2)	10 (19.6)	188 (19.1)	**18.6**
2+ No. (%)	75 (52.4)	107 (35.0)	1185 (41.5)	2736 (43.6)	308 (33.9)	19 (37.3)	384 (39.0)	**41.7**
**Therapy (%)**								
Insulin	14.0	13.4	12.0	11.9	10.6	15.7	13.1	**12.0**
Oral	42.7	41.5	41.5	44.4	36.6	29.4	44.2	**42.9**
Insulin/oral	10.5	13.1	11.8	11.8	13.0	25.5	15.3	**12.3**
None	32.9	32.0	34.6	31.9	39.9	31.4	27.4	**32.8**
**No. of deaths; Incidence rate of death per 1000 p-y (95%CI)**	96; 1.63 (1.33–1.99)	122; 0.58 (0.48–0.69)	939; 0.40 (0.37–0.42)	3087; 0.76 (0.73–0.79)	310; 0.40 (0.36–0.45)	12; 0.20 (0.12–0.36)	462; 0.67 (0.60–0.74)	**5028;** 0.61 (0.59–0.63)
**with Prior Hospitalizations for Diabetic Foot**								
**No. (%)**	39 (0.3)	86 (0.8)	1561 (13.9)	7049 (62.7)	1273 (11.3)	113 (1.0)	1125 (10.0)	**11,246**
**Mean Age yr (SD)**	71 (13)	69 (14)	74 (9)	75 (10)	69 (13)	67 (14)	73 (11)	**74 (10)**
**Male Sex (%)**	31 (72)	54 (60)	1099 (71)	4822 (64.8)	739 (58.1)	63 (55.8)	629 (55.9)	66.1
**Charlson index (%)**								
0 No. (%)	11 (25.6)	23 (25.6)	70 (4.5)	440 (6.2)	185 (14.5)	14 (12.4)	44 (3.9)	**7.0**
1 No. (%)	11 (25.6)	23 (25.6)	284 (18.3)	1122 (15.9)	287 (22.6)	21 (18.6)	183 (16.3)	**17.2**
2+ No. (%)	21 (48.8)	44 (48.8)	1199 (77.2)	5487 (77.9)	801 (62.9)	78 (69.0)	898 (79.8)	**75.8**
**Therapy (%)**								
Insulin	16.3	21.1	14.2	21.4	25.6	30.1	34.7	**22.3**
Oral	37.2	35.6	42.6	37.8	31.8	23.0	27.7	**36.6**
Insulin/oral	9.3	13.3	9.9	16.3	17.9	18.6	22.8	**16.2**
None	37.2	30.0	33.3	24.5	24.7	28.3	14.8	**24.9**
**No. of deaths; Incidence rate of death per 1000 p-y (95%CI)**	22; 0.54 (0.35–0.81)	39; 0.30 (0.22–0.40)	818; 0.39 (0.37–0.42)	4320; 0.56 (0.55–0.58)	633; 0.35 (0.32–0.38)	52; 0.30 (0.23–0.40)	749; 0.72 (0.67–0.77)	**6633;** **0.51 (0.50–0.53)**

**Table 2:** Prevalence rates of hospitalizations for diabetic foot complications across the years 2011 to 2018 in Tuscany. Hospitalization rate for diabetic foot complications = −038 × incidence rate + 14.2.

Year	Ulcers	Infections	Gangrene	Charcot	Revascularizations	Major Amputations	Minor Amputations	Total
	No. (%)	No. (%)	No. (%)	No. (%)	No. (%)	No. (%)	No. (%)	No. (%)
2011	138 (8.5)	127 (7.8)	940 (57.7)	9 (0.6)	358 (22.0)	18 (1.1)	38 (2.3)	1628 (14.1)
2012	144 (9.3)	130 (8.4)	871 (56.3)	10 (0.6)	335 (2.6)	24 (1.5)	33 (2.1)	1547 (13.4)
2013	139 (9.4)	117 (7.9)	814 (55.3)	9 (0.6)	348 (23.7)	16 (1.1)	28 (1.9)	1471 (12.8)
2014	122 (8.5)	104 (7.3)	795 (5.6)	7 (0.5)	349 (24.4)	20 (1.4)	33 (2.3)	1430 (12.4)
2015	117 (8.5)	102 (7.4)	743 (53.9)	1 (0.1)	348 (25.2)	17 (1.2)	50 (3.6)	1378 (11.9)
2016	114 (7.8)	109 (7.4)	780 (53.3)	3 (0.2)	399 (27.2)	18 (1.2)	41 (2.8)	1464 (12.7)
2017	106 (7.8)	115 (8.4)	695 (50.9)	7 (0.5)	377 (27.6)	19 (1.4)	45 (3.3)	1364 (11.8)
2018	105 (8.4)	104 (8.3)	644 (51.6)	5 (0.4)	340 (27.3)	11 (0.9)	38 (3.0)	1247 (10.8)
P for trend	NS	NS	0.0001	NS	<0.0001	NS	0.007	0.0001

## Data Availability

Data are available on reasonable request with the permission of Agenzia Regionale Sanità Toscana, Florence, Italy.
